# Secondary school teachers’ experiences of supporting students with mental health issues: a systematic review and meta-aggregation of qualitative studies

**DOI:** 10.3389/fpsyt.2024.1396008

**Published:** 2025-11-03

**Authors:** Mining Liang, Grace W. K. Ho, Martin Christensen

**Affiliations:** ^1^ School of Nursing, The Hong Kong Polytechnic University, Hong Kong, Hong Kong SAR, China; ^2^ Clinical Nursing Teaching and Research Section, The Second Xiangya Hospital, Central South University, Changsha, China; ^3^ The Interdisciplinary Centre for Qualitative Research, The Hong Kong Polytechnic University, Hong Kong, Hong Kong SAR, China

**Keywords:** adolescents, mental health issues, qualitative research, secondary school, teachers

## Abstract

**Background:**

Mental health issues in adolescents is steadily increasing, mainly since the rise of social media, the concomitant effects of internet use and other problems associated with academic performance, familial abuse and bullying. Secondary school teachers are exposed to a wide range of adolescent behaviours, yet depression and anxiety appear to be those behaviours that are causing the greatest concern.

**Aim:**

This review aimed to appraise and synthesise qualitative studies examining secondary school teachers’ experiences of supporting students experiencing mental health issues.

**Methods:**

Between January 2007 and March 2022, a search was conducted across nine databases, namely Medline, CINAHL Complete, APA PsycInfo, Embase, ERIC, Web of Science, Scopus, CNKI (for Chinese sources), and Wanfang (also for Chinese sources). This review focused on qualitative studies and included, but was not exclusive to, phenomenology, grounded theory, ethnography, action research, and feminist research. The selection of studies adhered to the guidelines outlined in the Preferred Reporting Items for Systematic Reviews and Meta-Analyses. The JBI approach was utilised for data extraction, synthesis, and critical evaluation. Additionally, the ConQual approach was employed to assess the reliability of the findings.

**Findings:**

A total of 2516 papers were identified, but only eleven qualitative studies were selected for inclusion in this review. Based on the JBI critical appraisal checklist for qualitative research, these studies were rated as having moderate quality. The studies originated from various countries, including three from the USA, one from the UK, Canada, Italy, Norway, Ireland, South Lebanon, Kenya, and China. A common theme identified in these studies was the challenges teachers faced when supporting students with mental health issues. Five categories emerged from the papers reviewed: ‘ Experiencing the Challenges of mental health issues in the Classroom’, ‘Teachers’ understanding of mental health issues’, ‘Feeling at a loss and coping with compassion’, ‘Providing a supportive and close relationship beyond that of the role’, ‘Understanding the Conflicts Imposed on and by the Education System’.

**Conclusion:**

It is evident from this review that secondary school teachers experience difficulties and challenges in supporting students with mental health issues. This also has implications for school nurses who may be best placed to support both students and teachers alike. It is also recommended that further research be conducted in Asian communities because of the paucity of published work in this setting.

## 1 Introduction

According to WHO World Mental Health (WMH) Survey, many mental disorders begin in childhood-adolescence (1). Globally, one in seven 10-19-year-olds will experience a mental disorder, accounting for 13% of the global burden of disease in this age group (2). Depression, anxiety, and behavioural disorders are among the leading causes of illness and disability among adolescents (2). In the UK and the US, there has been a 5% increase in depressive symptoms over a 10 year period (3, 4) and a threefold increase in anxiety disorders, and self-harm (5). In addition, Burstein et al. (6) along with Haidt and Lukianoff (7) found that emergency department visits for suicide attempts and/or suicidal ideation has doubled among teenagers over an eight year period (2007 and 2015). Similarly, two recent Chinese national surveys, found that over 17% of adolescents had mental health issues (8, 9) and with the advent of the COVID-19 pandemic, Ma et al.’s, (10) systematic review found that the prevalence of depression and anxiety among young people was over 25% (range 26-29%). The reasons may be multi-factorial. However, the rise of social media activities and internet use is being viewed as one of the major reasons that has seen an exponential increase in adolescents with mental health issues, with at least 92% of teenagers active on social media at any one time (11). Activities such as repeated checking of messages, personal investment in terms of time spent, and addictive use of social media (leading to insomnia) have been positively correlated with increased levels of depression, anxiety and psychological distress in the adolescent age group (12, 13).

### 1.1 The increase in mental health issues among adolescents

In the 21st century, there have been several social changes that may have contributed to the steady rise in mental health burden in current generations of adolescents, one of which is the rise in the popularity of social media among adolescents since the advent of the smartphone (6, 7). For example, one longitudinal cohort design study in Iceland found that time spent on social media was associated with depressive symptoms and the physical symptoms of anxiety, especially for girls (14). Similarly, another cohort study in the UK found that when compared with 1 to 3 hours of daily use versus 3 to 5 hours led to a 5% increase in depressive symptoms. The authors also found that greater social media use was related to an increase in online harassment, poor sleep patterns, low self-esteem and poor body image, which in turn these were reflected in higher depressive symptom scores (15).

Of concern is that there is a threefold increase in the probability of developing a mental health problem in adulthood if mental health issues were present in adolescence (16, 17). For example one study found that half of the estimated 7.7 million US adolescents with a treatable mental health disorder did not receive needed treatment from a mental health professional (18). The long term of effects of this not only include the existing mental health issue, but the development of comorbid conditions such as obesity, cardiovascular disease, type II diabetes, and some cancers; resulting in significant decrease in life expectancy of up to 30 years (19, 20).

### 1.2 Schools as an important developmental context for adolescents

According to Bronfenbrenner’s (21) ecological model, schools (along with other microsystems, including the family) are the most immediate developmental context for adolescents, and schools can exert the greatest influence on their development such as peer relationships, social interaction, academic achievement, cognitive progress, emotional control, behavioural expectations, and physical development are all involved (21, 22). Schools play a crucial role in adolescent mental health, from providing an excellent setting for targeting children’s mental health issues, developing and supporting their academic performance, as well as the important social connections they develop. Often the resulting mental health issues experienced by adolescents, apart from those attributed to social media and internet use, stem from educational failure (23) or those who have been excluded from school (24).

### 1.3 Teachers’ role in supporting students’ mental health

As teachers spend a significant amount of time with students, they are well-placed to notice symptoms and behaviour of anxiety, depression, and other mental health issues (25). Moreover, since teachers are often the first contact point for parents and other psychological professionals within schools, they are ideal people to refer students to mental health services (26). Investigating the correlation between teacher-student relationships and depression among adolescents, studies have found that students whose teacher-student relationships declined over time reported the greatest increase in the incidence of depression, in contrast, students who reported an increase in the teacher-student relationship had lower levels of depression during this same timeframe (27, 28). Moreover, some studies have also indicated that increased teacher support moderated the effect of stress on externalising problems (27, 29), however, those students who had issues with internalised problems, and level of teacher interaction made no difference in levels of student depression (29). Additionally, one study has examined the role of adults outside of the family who can serve as stress moderators (30). Teachers are most frequently reported as positive role models outside of the family. Role models outside of the family allow children to identify with adults they respect, look up to, and admire. Therefore, besides promoting academic achievement, teachers provide children with positive role models of self-identification (29). Therefore, as a result, teachers can play a vital role in buffering the effects of stress by providing care, concern, fairness, respect, and empathy for those students more vulnerable to stressful situations.

### 1.4 Teacher’s response to their students’ mental health issues

With the increase in mental health issues in adolescents, teachers have found a wide range of diagnosed and undiagnosed mental health problems in the classroom which require them to support or at least address (31). Although teachers have identified mental health issues as one of the greatest healthcare needs for their students (32), a recent mixed-method study highlighted teachers had a mismatch between feeling responsible for and being able to help students experiencing a mental health issue (33). This is evident in a recent systematic review which provided strong evidence of the importance school climate has in influencing an adolescent’s mental health, and pointed out that schools can promote adolescent mental wellness by addressing the psychosocial school climate while utilising the expertise and skills of their teachers (34). This is an important consideration where teachers play an important role in addressing the mental wellbeing of adolescents across all levels of prevention. Yet, teachers feel ill-equipped to deal with the challenges that a student with a mental health issue poses especially when the student is depressed or over anxious.

For example, the results of an online Canadian survey, found that 36% of secondary school teachers felt confident in their knowledge of mental health issues when supporting students experiencing these issues, whereas only 26% felt they had the necessary knowledge and skills (35). This has been likened to a form of mental health literacy, albeit at varying levels, which has been defined by Jorm, et al. (36) as knowledge and beliefs about mental disorders that aid in recognition, management, or prevention. The main components of mental health literacy, include knowledge of mental health issues, stigma towards mental health issues, confidence in helping others, and behaviour of helping others (37). Miller, et al. (38) found that levels of high school teacher depression literacy was significantly associated with high school student depression literacy, meaning if teachers fully understood what depression and/or anxiety is, how it is managed and supported, then this would be reflected in the level of understanding among the students they taught. However, this a reverse of what actually happens in reality and therefore, what this amounts to is a form of mental health illiteracy among these teachers, suggesting that most secondary school teachers are unable to recognise mental health issues among their students. Miller, et al.’s, finding highlights the importance of optimising teacher depression literacy in order to maximise student depression literacy.

Yet, a number of studies have highlighted that mental health literacy overall is poor among secondary school teachers (35, 39–42). This is exemplified in a recent cross-sectional study with 304 secondary school teachers in Tunisia, where Fekih-Romdhane et al. (41) found that teachers’ overall lack of knowledge of mental health issues, held negative attitudes towards schizophrenia, and 41% felt uncomfortable with the student displaying symptoms of schizophrenia. Additionally, Yamaguchi et al. (42) survey using depression and schizophrenia vignettes found that Japanese high school teachers were able to correctly recognise depression in 54% and schizophrenia in 35% of cases, with less than 20% of teachers’ having the confidence to help depressed students. Within the Chinese context, there are very few studies that have examined secondary school teacher’s knowledge about mental health issues. However, a recent national survey found that mental health literacy among the Chinese adult population is very poor (43). This suggests that secondary school teachers’ in China may have insufficient knowledge and skills about mental health issues, especially if the wider Chinese population has little knowledge of mental health issues, its diagnosis and management more broadly.

### 1.5 Teacher training and education

It is perhaps this lack of knowledge and skills which prevents teachers from supporting students with mental health issues, and therefore it, would seem important to ensure that teachers have adequate levels of knowledge, the confidence and skills in order to reduce the impact of mental health issues on students. This is evident from the findings of two recent systematic reviews conducted to examine the effectiveness of mental health training programs on knowledge, attitudes, or helping behaviour of secondary school teachers’ (44, 45). Anderson et al. (44), for example found that while all eight included studies reported improvement in secondary teachers’ knowledge and attitudes about depression, anxiety and related mental health issues in adolescents post intervention, there was very little evidence that the included mental health training programmes improved teachers’ helping behaviours or students’ mental health overall.

While it can be accepted that mental health training might be useful, the challenge is finding the right or appropriate approach for teachers. For example, mental health training programs can be understood through the classical framework of behaviour change, according to which an increase in knowledge and positive attitudes leads to a change in behaviour (46). In the context of teacher training, this theory states that improving teachers’ knowledge and attitudes will lead to a greater frequency of helping behaviours toward students. A more recent systematic review by Mo, et al. (47) which focused on the effectiveness of school-based gatekeeper training for suicide prevention, concluded that gatekeeper training appears to have the potential to change participants’ suicide prevention knowledge and skills, but that more studies of better quality are needed to determine the effectiveness in changing gatekeepers’ attitudes.

An initial search of four databases (the Cochrane Database of Systematic Reviews, the JBI Evidence Synthesis, MEDLINE and PROSPERO) was undertaken to determine whether any systematic reviews qualitative or quantitative are currently being investigated on the topic. Indeed, much of the published literature focuses on cohort and control group studies using recognised scales such as survey designs eliciting data concerning the more quantitative characteristics of mental health issues among students especially those in higher education. Yet, there are very few published studies that had as its focus the qualitative experience of supporting mental health issues from the teacher’s perspective and less so from the secondary school sector. Moreover, a paucity of qualitative reviews makes it difficult to determine the current problems voiced by teachers who try to support secondary school students that are mentally unwell. Therefore, the aim of this review was to appraise and synthesise qualitative studies examining secondary school teachers’ experiences of supporting mentally unwell students so as to better understand the difficulties and complexities that confront them, for example 1) the emotional impact of supporting a mental unwell student, 2) how they interact with the education system around mental health issues and 3) the benefits of training and education. Moreover, it may offer insights as to how teachers’ experiences may help school nurses and other healthcare professionals to gain a deeper understanding about teacher needs together with the development of secondary school-based education programmes to help teachers better care for students with mental health problems.

## 2 Aim

To appraise and synthesise the findings from qualitative studies examining the experiences of supporting students with mental health issues among secondary school teachers.

## 3 Review question

Currently, there are very few systematic reviews that specifically discuss secondary school teachers’ experiences in supporting students with mental health issues. Therefore, this review aims to establish what is known qualitatively about secondary school teachers’ experiences and mental health issues in the classroom. Based on the PICo (Population, Phenomena of Interest & Context) – P: school teachers, I: experiences of mental health issues in the classroom, and C: secondary schools, the review question was: “What are the experiences of secondary school teachers in supporting students with mental health issues?”

## 4 Materials and methods

### 4.1 Search strategy

Search terms in the review question were identified using the PCC framework (population, concept, and context). Secondary teachers (including school teachers and secondary school & high school teachers) were searched along with terms related to mental disorders and qualitative research methods. The search strategy employed in this review consisted of three steps. The first step was to conduct a limited search of MEDLINE (PubMed), CINAHL (EBSCO), and CNKI (in Chinese) to identify articles relevant to the topic. An analysis of the titles and abstracts of relevant articles, as well as the index terms used to describe the articles, was conducted to develop a comprehensive search strategy. As a second step, we used the adapted full search strategy to search nine databases (Medline, CINAHL Complete, APA PsycInfo, Embase, ERIC, Web of Science, Scopus, CNKI (in Chinese), and Wanfang (in Chinese)) from January 2007 to March 2022. The dates correspond with the rapid escalation of anxiety and depression in secondary school children with the advent of the smartphone and the rise in popularity of social media such as Facebook (7). We included all studies that met our inclusion criteria. In addition, we considered grey literature such as conference proceedings, unpublished commentaries, and discursive articles. Lastly, to identify additional studies, we hand-searched the reference lists of all studies identified for critical appraisal (**Table 1**).

**Table 1:** An example of search strategy (Medline).

Search ID	Search Terms	Results
S1	((MH “Qualitative Research”) OR (MH “Empirical Research”)) OR (“qualitative stud*” OR “empirical research” OR “qualitative research” OR “qualitative method” OR “case stud*” OR interview OR “Empirical Research” OR “behaviour research” OR “comparative study” OR “observational study” OR “personal narrative”)	720,049
S2	(MH “mental health” OR MH “mental disorders”) OR (“mental disorders” OR “mental health” OR “mental health problem” OR “mental health issue” OR “mood disorder” OR “bipolar and related disorder” OR “anxiety disorder” OR schizophrenia OR “self-harm” OR suicide)	731,188
S3	(MM “School Teachers”) AND ((secondary OR high))	507
S4	(“high school” OR “secondary school”) AND (teacher OR educator)	3,953
S5	S3 OR S4	4,304
S6	S1 AND S2 AND S5	68

### 4.2 Inclusion and exclusion criteria: participants and phenomena of interest

Based on the PICo (Population, Phenomena of Interest & Context) – P: school teachers, I: experiences of mental health issues in the classroom, and C: secondary schools, the review question was: *“What are secondary school teachers experiences of supporting students with mental health issues?”* For this review participants had to be secondary school teachers providing education for adolescents between the ages of 12-18. If the study included teachers for students both within and outside of this age range, then studies were only included if findings unique to students in question could be determined and extracted. Those studies that included mental health professionals, such as guidance counsellors or school-based mental health providers, were excluded from the review as did those studies that only included primary or tertiary school teachers. In this review, we reviewed qualitative studies that examined teachers’ experiences supporting students with mental health issues. Other phenomena such as teachers’ experiences of working with other professionals, parents’ experiences, and teachers’ needs were excluded. Studies examining teachers’ experiences in general mental health promotion/supporting mental health of students in schools were also excluded. This review focused on qualitative studies, including, but no inclusive of, phenomenology, grounded theory, ethnography, action research, and feminist research. Mixed methods studies with qualitative data were included if the qualitative data was easily accessible and recognisable. Papers must provide a qualitative account using recognised qualitative methods of data collection in order to be considered for inclusion, face-to-face interviews or focus groups, for example. Articles must use qualitative methods to analyze their data, such as content, thematic, or narrative analysis, for inclusion in the data synthesis. The following types of articles were excluded if they were quantitative studies, empirical approaches, discussions, discursive, literary, or quantitative systematic reviews, and articles not in English and/or Chinese. In this systematic review, we used the Joanna Briggs Institutes System for the Unified Management of the Assessment and Review of Information (SUMARI) and followed the Consolidated Criteria for Reporting Qualitative Research (COREQ) (48).

### 4.3 Study selection

**Figure 1:**
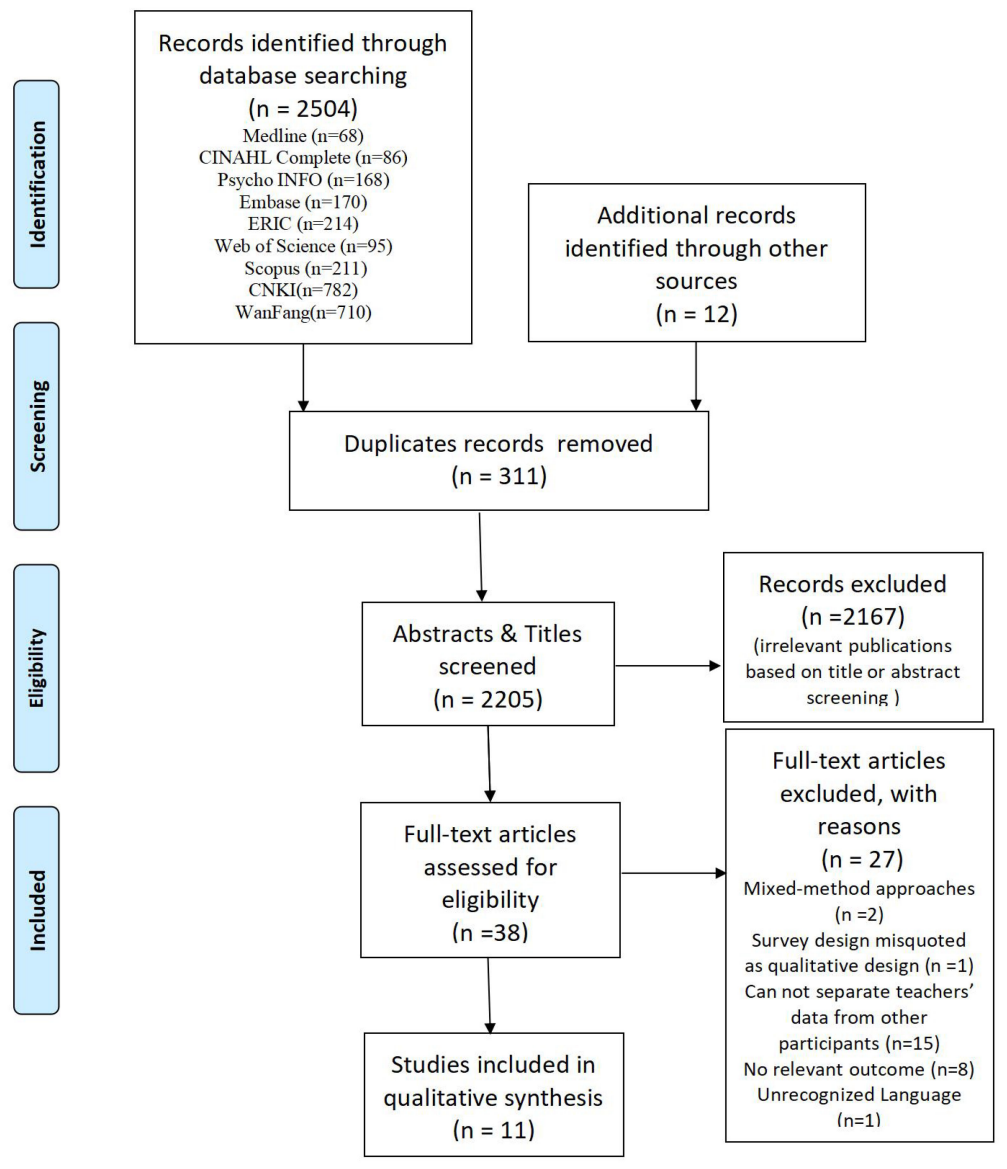
PRISMA flow chart of article selection, screening, and assessment (49).

All articles identified by search strategy were compiled and uploaded to EndNote X9, with duplicates removed. Title and abstract submissions were reviewed against the inclusion criteria. Once the selected studies were identified, full texts were obtained and reviewed independently using JBI’s SUMARI framework for appraising qualitative papers. At the completion of the independent review, discussions were held to resolve any disagreements. A total of 2516 articles were found. After screening and further refinement to the inclusion criteria, 11 were included in the final synthesis (**Figure 1**).

### 4.4 Assessment of methodological quality

Two independent reviewers critically appraised the eligible studies using the standard JBI Critical Appraisal Checklist for Qualitative Research within the SUMARI programme. Based on the 10-item critical appraisal checklist, reviewers rated each paper yes, no or unclear based on methodological and philosophical soundness. Each paper was assessed using criteria that established whether the following items: Q1 (Congruence between philosophical perspective and research methodology), Q2 (Congruence between research methodology and research questions/objectives), Q3 (Congruence between research methodology and data collection methods), Q4 (Congruence between research methodology and data presentation/analysis), Q5 (Congruence between research methodology and interpretation of results), Q6 (Cultural or theoretical situatedness of the researcher), Q7 (Address the researcher’s influence on the research, and vice versa), Q8 (Ensure participants’ voices are adequately represented), Q9 (Research ethics), Q10 (Conclusions drawn in the research report). Data that is missing would be requested from authors of papers for clarification. Any disagreements between the reviewers were resolved through discussion (ML and MC).

### 4.5 Data extraction

The JBI data extraction tool was used by two independent reviewers (ML and MC) to extract data from included studies (50). A wide range of data was extracted, including details about the study populations, the context, the culture, the geographical location, the methods, and the phenomena of interest. We extracted the findings and illustrated them verbatim and gave them a level of credibility. Disagreements between the reviewers (ML and MC) were resolved by discussion or with a third reviewer (GH). Included studies did not require further information from the corresponding author (**Table 2**).

**Table 2:** Characteristics of included studies.

Study	Methods for data collection and analysis	Phenomena of interest	Setting/context/culture	Participant characteristics and sample size	Description of main results
Buchanan and Harris (51) Canada	Data Collection: interviews and journaling Analysis: Thematic	1. To identify what teachers need for themselves when a student attempts suicide and is then subsequently returned to their classroom. 2. To identify teachers’ views on what they need to be able to effectively help students feel connected, valued, and safe.	Junior high or high schools in Newfoundland.	6 teachers	Themes include: the many hats of the teachers; Thoughts and feelings of shock, sadness, uncertainty, anxiety and fear; Coping: a feeling of relief; Confidentiality: to tell or not to tell
Dirks (52) USA	Data Collection: interviews, field notes of classroom teaching and environmental observation Analysis: Thematic	To examine the phenomenon of adolescent anxiety and depression through the lived experiences of novice and experienced high school music educators.	High school	10 novice and experienced high school music educators	Warning signs, Causes, Student help-seeking behaviours, Responses, Impact on music educators, Social-emotional support within the teaching environment
Doumit, et al. (53) Lebanon	Data Collection: Focus groups Analysis: Thematic	To identify knowledge, attitudes, and practices of teachers and parents concerning child/adolescent mental health.	Elementary, middle, and secondary levels in two private hub schools	27 teachers and 18 parents	Mental health care is a priority for overall health, mental health issues is a cultural taboo, There is a need for better education and cultural understanding about mental health
Greif-Green, et al. (54) USA	Data Collection: interviews Analysis: Grounded Theory	To explore how teachers conceptualise mental health needs among youth.	Middle and high school teachers from across the United States	29 teachers	Identifying emotional and behavioural challenges
Iudici and Fabbri (55) Italy	Data Collection: interviews Analysis: Content	How teachers report psychological discomfort of the students and on what criteria does their procedure?	A middle school	42 teachers	Identifying, reporting mental health issues
Maelan, et al. (56) Norway	Data Collection: interviews and focus groups Analysis: Thematic Processing	To explore teachers’ and head teachers’ understandings of how they work to support pupils’ mental health through their everyday practices	10 secondary schools for individual interviews and 4 schools for focus group	10 head teachers participated in individual interviews and 36 teachers participated in the six focus groups	Relationship between teachers and pupils, Strategies used to reduce pressure and anxiety, Develop an inclusive school climate, Provide experiences of mastery and different learning opportunities
Mbwayo et al. (57) Kenya	Data Collection: focus group Analysis: Unclear	To seek teachers’ perceptions of mental health concerns that are relevant in school settings	Secondary school	60 public primary and 50 secondary school teachers	Identify mental health issues, Lack of skill and time
McConnellogue and Storey (58) Ireland	Data Collection: interviews Analysis: IPA	To explore the subjective experiences and perceptions of teachers in a school-based suicide prevention role in depth.	A large single-gender secondary school	7 teachers	Baseline mastery, Threats to efficacy, tension between personal and professional identities
Shelemy et al. (25)	Data Collection: interviews Analysis: Thematic	To discover the lived experiences of math and language arts teachers as they educate high school students in high-poverty rural schools affected by anxiety.	High school	8 teachers	Teachers perceived role, Relationship building, Finding balance, The learning environment, Changes in the classroom, Background knowledge and training, Positive perceptions of the school counsellor
Strasser (59)	Data Collection: interviews Analysis: IPA	To explore teachers’ perspectives of supporting students’ mental health, focusing on their emotional and cognitive processing of these experiences.	Secondary state schools in the South East and London regions	7 teachers	Perceived role of teacher, Nature of relationship, Barriers to helping the child, Amount of training and resource, Helplessness and satisfaction
Yao et al. (60)	Data Collection: interviews Analysis: Content	To identify how Ban Zhu Ren perceive the mental health of their students, and how they have acted on these perceptions.	Middle schools (ages 12 to 15) in Zhejiang and Anhui provinces	27 head teachers	Informant perception of the prevalence of mental health issues among adolescents; Informant classification of student mental health issues; Informant understanding and labelling of student behavioural issues; Ways that informants determine mental health issues among their students; Informant interventions for students they believe to have mental health issues; Informants’ perceived ability to intervene with student mental health issues; Training informants received on intervening with student mental health issues

A finding was defined as a literal extract of the authors’ analytical interpretation. For consistency, all studies extracted findings at the same level (e.g. themes, categories). To illustrate meaning, each finding was accompanied by an illustration that included quotes in the voice of the participants. Each findings credibility was appraised as “unequivocal (U),” “credible (C),” or “unsupported” (50). Unequivocal findings were accompanied by illustrations that were beyond a reasonable doubt (participant direct quote). Credible findings were accompanied by illustrations that were not clearly linked to the findings and therefore could be challenged (author suppositions). Unsupported findings were not supported by illustrations and were not included in the data synthesis (50).

### 4.6 Data synthesis

Based on the meta-aggregation of JBI SUMARI, qualitative findings were pooled into categories. The findings and illustrations of the text were identified after reading it several times. We included only unequivocal and credible findings in our synthesis. The findings were grouped into categories based on their similarity in meaning. These categories were then synthesised to produce summary findings that could be used to inform evidence-based practice. To reach consensus, the first reviewer (ML) drafted these, and then discussed and revised with the other reviewers (MC and GH).

### 4.7 Assessing confidence in the findings

Using the ConQual approach, the final synthesised findings were graded for their confidence in their output of qualitative research synthesis for the tenets of qualitative rigor (credibility and dependability) (61). Each synthesised finding was classified as high, moderate, low, or very low in terms of confidence. Each qualitative study was graded as high quality at the outset, and then each synthesised finding was downgraded on the basis of dependability and credibility. A ConQual score was used to rate the confidence in the synthesised findings (**Table 3**).

**Table 3:** ConQual score to determine the level of dependability and creditability.

Systematic review title: The experience of secondary school teachers in supporting students with mental health issues Population: Teachers Phenomena of interest: Supporting students with mental health issues Context: Secondary schools				
Synthesised Finding	Type of Research	Dependability	Credibility	ConQual Score
The challenges of supporting the students with mental health issues	Qualitative	Downgrade 1 level	Downgrade 1 level	Moderate

Dependability: Downgraded one level three studies (3/11) scored 5, seven (7/11) scored 3, and one (1/11) scored 1 for the dependability questions.

Credibility: Downgraded one level due to a mix of U and C findings. U =52, C = 2.

### 4.8 Study inclusion

Among 2516 eligible articles, 2504 possible articles were located from the nine databases and 12 articles were retrieved by hand. 2205 articles (759 English, 1446 Chinese) were screened by title and abstract after 311 duplicate articles were removed, resulting in the exclusion of 2167 articles that did not meet inclusion criteria. There were 38 articles (32 in English, 6 in Chinese) retrieved for full-text review. In addition, a further 27 studies were excluded since two studies used mixed-method approaches, one study was a survey design misquoted as a qualitative design, 15 studies could not separate teacher’s data from other participants, such as guidance counselors, eight studies had no relevant outcome, and one study was in a language other than English and/or Chinese (**Figure 1**).

### 4.9 Methodological quality

Using the JBI Critical Appraisal Checklist for Qualitative Research, two studies were of low quality with scores of 4/10 (one study) and 5/10 (one study); five studies were of medium quality with scores of 6/10 (two studies), 7/10 (three studies); four studies were of high quality with scores of 8/10 (one study), 9/10 (two studies), and 10/10 (one studies). For the purposes of this review, studies that scored 7 or above were considered of good to excellent quality, while those below were considered low quality. All papers were included in the final meta-aggregation because the findings from each provided an overall picture of teacher’s experiences of mental health issues in classroom (**Table 4**).

**Table 4:** Critical appraisal of included qualitative studies.

Study	Q1	Q2	Q3	Q4	Q5	Q6	Q7	Q8	Q9	Q10	Score
(51)	Y	N	Y	Y	Y	N	Y	Y	N	Y	7
(52)	Y	Y	Y	Y	Y	Y	Y	Y	Y	Y	10
(53)	U	Y	Y	U	Y	U	N	Y	Y	Y	6
(54)	Y	Y	Y	Y	Y	U	N	Y	Y	Y	8
(55)	U	Y	Y	U	U	N	N	Y	Y	Y	5
(56)	U	Y	Y	N	Y	U	N	Y	Y	Y	6
(57)	U	Y	U	N	U	N	N	Y	Y	Y	4
(58)	Y	Y	Y	N	Y	N	N	Y	Y	Y	7
(25)	Y	Y	Y	N	Y	Y	Y	Y	Y	Y	9
(59)	Y	Y	Y	Y	Y	Y	Y	Y	N	Y	9
(60)	N	Y	Y	Y	Y	N	N	Y	Y	Y	7

Y, yes; N, no; U, unclear;

Q1 Is there congruity between the stated philosophical perspective and the research methodology?

Q2 Is there congruity between the research methodology and the research question or objectives?

Q3 Is there congruity between the research methodology and the methods used to collect data?

Q4 Is there congruity between the research methodology and the representation and analysis of data?

Q5 Is there congruity between the research methodology and the interpretation of results?

Q6 Is there a statement locating the researcher culturally or theoretically?

Q7 Is the influence of the researcher on the research, and vice-versa, addressed?

Q8 Are participants, and their voices, adequately represented?

Q9 Is the research ethical according to current criteria or, for recent studies, is there evidence of ethical approval by an appropriate body?

Q10 Do the conclusions drawn in the research report flow from the analysis, or interpretation, of the data?

Six studies met the criterion for Q1 (congruity between stated philosophical perspective and research methodology), with five studies employed a qualitative descriptive approach without clearly stating a philosophical orientation. Ten studies met the criterion for Q2 (congruity between research methodology and research questions/objectives), with one study gave a set of prescriptive questions, which was incongruence with the phenomenon methodology. Ten studies met the criterion for Q3 (congruity between research methodology and data collection methods), with one study used focus group discussions to collect data, which was incongruence with the phenomenon methodology. Five studies met the criterion for Q4 (congruity between research methodology and data representation/analysis), with six studies’ data analyses were incongruent with the phenomenon methodology. Nine studies met the criterion for Q5 (congruity between research methodology and interpretation of results), with two studies’ interpretation of results were incongruent with the phenomenon methodology. Three studies met the criterion for Q6 (statements locating the researcher culturally). Four studies met the criterion for Q7 (the influence of the researcher on the research and vice versa). Eleven studies met the criterion for Q8 (representation of participants and their voices). Nine studies met the criterion for Q9 (evidence of ethical approval), although two studies did not provide information on ethical approval by an appropriate body, we decided to include them since vulnerable participation and anonymity were implied from the text. Eleven studies met the criterion for Q10 (conclusions flow from the data analysis/interpretation).

### 4.10 Characteristics of included studies

The eleven studies included in this review were published between 2014 and 2018. The studies took place in nine countries: three from the USA, one from the UK, one from Canada, one from Italy, one from Norway, one from Ireland, one from South Lebanon, one from Kenya, and one from China. The total sample size was 283 participants. All studies used qualitative research designs. Eight of the eleven studies collected data through in-depth, semi-structured interviews, two through focus groups, and one using both. A variety of data analysis methods were used including thematic and content with some studies adopting a phenomenological approach (either descriptive of interpretative).

## 5 Review findings

Overall, teachers experienced considerable challenges when supporting students with mental health issues. The one synthesised finding was based on five categories, which were supported by 51 findings. Five categories emerged from the papers reviewed: *‘Experiencing the Challenges of Mental Health Issues in the Classroom’, ‘Teachers’ Understanding of Mental Health Issues’, ‘Feeling at a Loss and Coping with Compassion’, ‘Providing a Supportive and Close Relationship beyond that of the Role’, ‘Understanding the Conflicts Imposed on and by the Education System’.*

### 5.1 Category one: experiencing the challenges of mental health issues in the classroom

Category one described the experiences of teachers when they faced a student who had a mental health issue. Secondary school teachers stated that mental health issues were common and there had been an increase in the numbers among adolescents they saw in the classroom (60). They experienced at first hand the behaviours exhibited by their pupils, for example, the physical signs of anxiety and depression including crying and verbal outbursts, emotional withdrawal behaviours, and they also noticed anxiety levels increased significantly when the students had an upcoming test or exams (52, 54, 57, 59). Conversely, secondary school teachers also discussed mounting academic pressures, adverse family events such as divorce, sexual abuse, traditional and cyberbullying, gender transformations, and immigration as being additional causes of some of the mental health issues they encountered (52).

### 5.2 Category two: teachers' understanding of mental health issues

Category two describes secondary school teachers’ understanding of mental health issues and how these might cause discomfort for the student experiencing challenges in their lives. Yet, being to adequately define or describe what they saw as a mental health issues was difficult. For example, secondary school teachers in Italy had difficulty defining what ‘psychological discomfort’ meant and how they would recognise it against a backdrop of other typical adolescent behaviours (55). Conversely, secondary school teachers in US used a wide variety of markers to identify students with mental health issues (54). This proved troublesome because some teacher-derived markers were not included in standardised assessments such as a change in student behaviour, tracking the duration of the student’s problems, or observing students crying, inappropriate reactions to teachers, and spontaneous acting out (54). Therefore, secondary school teachers mostly relied on previous experiences as a teacher and/or parent to identify students with mental health issues (54). However, secondary school teachers in Lebanon highlighted that mental health is not a common topic generally and that consideration towards those with a mental health problem is seen as a culture taboo, so much so that the fear of being labelled as “mentally ill” often prohibits teachers identifying students as having a mental health issue out of concerns for the parents (53). In addition, vague behaviours such as ‘tired of learning’, ‘being rebellious’, or ‘falling in love’ at a young age, while not considered to be a symptom of mental health issues by mental health professionals, have, however, been established as signs of mental health issues by secondary school teachers and as such appear to have been normalised by some teachers as self-harm (60).

### 5.3 Category three: feeling at a loss and coping with compassion

It is in this category that secondary school teachers felt at a loss as how to help their students and their inability to cope with the mental health issues experienced by their students. In most cases the teachers expressed a deep sense of compassion for their students who they saw as struggling with their mental health issues. It is here that feelings of sadness, anxiety and worry pervaded the teachers’ minds. Teachers initially expressed shock when they were first informed of mental health issues, believing that such things were unlikely, for example, *“I did not know the student even had emotional problems or issues at all.”* (51). Sadness was often a reaction to the news of a student with a mental health issue (25, 51), which create feelings of anxiety or worry especially when they didn’t what to do and the uncertainties of not knowing what the signs or symptoms they should be looking for (51). Teachers also expressed fear, being afraid they might aggravate student’s behaviour to the point where a suicide attempt could be become a reality (51). Meanwhile, teachers felt helpless and frustrated when they confronted barriers to services which would otherwise help the students (60) - *“There was nowhere I could put her, there was nowhere private I could take her, so it was just very frustrating the kind of mental health support we were offering.”* (25, p337). Moreover, teachers felt a great sense of pride when they made a positive change towards the student’s well-being, especially in terms of academic achievement and personal development. One teacher commented that her effort was paying off because as she described it the student was seeing the light at the end of the tunnel - *“I just felt so pleased that I did it [helped]. I said to my daughter in the car on the way home it was the right thing to do [ … ] I just felt elated that he was coming out the other end”* [(25); p377]. Additionally, teachers also felt a great deal of relief when they found someone to look after the student more professionally, someone who was better qualified to handle mental health issues than them, which for the teachers clearly demonstrated their own limitations in supporting the student (51, 59).

### 5.4 Category four: providing a supportive and close relationship beyond that of the role

Category four demonstrated that the role of the teacher had expanded beyond simply being their teacher, but to one of forming and developing close relationships between themselves and the student, which for many they saw as being particularly important in supporting the student’s mental health. It also describes the difficulties and challenges caused by this supportive relationship. First, secondary school teachers reported that their role had now encompassed more than simply teaching the curriculum, but also now included being an advocate, a surrogate parent or a resource for those students struggling with a mental health issues (51, 59). However, there were some secondary school teachers who may have been hesitant to accept a role in supporting mental health issues due to what they felt were the expectations of teachers (25, 58), for example: *“It’s not our responsibility. I think we’re not trained to be counselors we should … send them off, refer them to someone else cause we can’t take responsibility. That’s what I feel”* [(25); p375].

Second, teachers argued that having a supportive relationship with students together with an open-door policy would increase help-seeking behaviour (25) and encourage students to ‘drop by’ (52). Teachers expressed that forming these close supportive relationships allowed for the opportunity to share experiences and to build trust (52, 56), which in turn would allow the student to feel comfortable and more fully engaged without fear of feeling excluded or marginalised because they were struggling mentally (25).

However, some teachers worried about the blurring of the boundaries in the teacher/student relationship and were concerned with the consequences of becoming too close (25). This produced some tensions between personal and professional identities (58) and this is especially important where the conflict around what constitutes the teachers’ role may have contributed to the resistance of having an adjunct role in supporting those students with a mental health issues - *“You know we’re not psychiatrists. We … our role is to identify and pass on as quickly as possible”* (58). This created additional challenges specifically around student confidentiality. Some teachers felt that confidentiality should be applied consistently and a student’s right to privacy should be upheld regardless, while others liked knowing more about the student’s situation (51). In addition, some teachers found it difficult to maintain confidentiality and honesty, either because they were overwhelmed by the student’s situation and needed guidance, or because they weren’t sure what actions were needed to help them (51).

### 5.5 Category five: understanding the conflicts imposed on and by the education system

The last category describes the teachers experiences of trying to provide a supportive environment will navigating the policies and procedures governing mental health issues in the classroom. First, secondary school teachers expressed the impact of mental health issues on their teaching practice or how they supported students with mental health issues within the school context. For example, secondary school teachers became much more aware of the possibility that students could be experiencing some form of mental health crisis and made the decision they would make the school environment as safe and welcoming as they could (25, 51, 52, 59). In some instances secondary school teachers allowed for and actively encouraged the discussion of sensitive topics such as life themes and mental health issues, and by doing so were able to promote conversations about resiliency and recognising depression as a means to reducing the stigma associated with mental health issues (56, 59). This was important to minimise the effects of mental health issues while providing a conducive learning environment, one in which the student/s felt able to contribute and be part of - “*The classroom climate is one of the most important things. It can both promote and undermine mental health … there is, for example, zero tolerance for negative comments, so that everyone can be who they are and feel safe”* [(56), p22]. Moreover, some teachers provided students with different learning opportunities, for example, making adjustments to content delivery to facilitate student learning or provided extra-curricular activities, again activities to promote inclusivity (52, 56).

Sadly, some teachers suggested they should simply refer students to and build bridges with mental health professionals when they need more help than just a listening ear, such as including counseling teams, administrators, and parents (25, 52). However, some teachers felt unsupported by the school and/or parents - such as teachers not having the time to follow up with the students after counseling, or overburdened administrators and counseling departments, or parents who didn’t want it known their child had a mental health issues, or a lack of feedback from parents and mental health professionals outside of the school system (25, 57, 58). Teachers also noted the difficulties raised by the education system itself inasmuch that teachers were frequently overwhelmed by the large number of students and therefore were unable to give the time and effort required to help those students struggling mentally (53, 55, 59).

Teachers also expressed a lack of knowledge or training about mental health issues, one commenting on the divide between academic achievement and having an awareness of adolescent behaviour - *“Most seminars given to teachers are on academics not on emotional or behavioural problems of children”* [(57), p159]. Consequently, teachers didn’t know what the signs or symptoms were, what they should be looking for, what to do, and if there was a right thing to do (51, 52, 60). Yet, even though some teachers had received mental health training, participants felt that training resources provided to them were less than adequate (25) with one commenting that it was ‘useless’ and feeling less informed than before attending the course - *“I don’t think training helps much, because the content is theoretical and it does not teach us how to work with specific mental health issues, and the training is not all about adolescent mental health. It is useless when we have problems with students”* [(60), p11].

## 6 Discussion

The challenges teachers experienced while offering support to mentally unwell students in the classroom were unprecedented. Many participants described their emotional turmoil and internalised feelings at having students experiencing significant mental health issues at such a young age. It is clearly evident that teachers are completely unprepared for supporting mentally unwell students, not knowing what to do and what level of support students required or needed or simply possessing a lack of understanding about mental health issues generally (35, 62). Yet, this created a dilemma between needing training and supporting the student. Asking teachers to assess their students for signs of stress or depressions, Wyman, et al. (63) found that many of their participants were reluctant or unprepared to initiate a conversation with a student who was experiencing some mental health challenges for fear of this leading to a suicide attempt or moreover, talking candidly about previous suicide attempts. While many teachers expressed a need to be more informed about mental health issues, the reality of training and education was often controlled by school administrators who were more in favour of referring students to mental health professionals instead of attempting to address the problem directly in the classroom (64). This often led to feeling unsupported not only by school administrators but with counselling services or even the student’s parents, which clearly impacted on the ability of the teacher to support mentally unwell students (65–67). The reverse of this were the teachers feeling helpless and overly burdened to the point that it started to affect their own mental and physical well-being and conversely, their ability to work effectively (66).

One way the teachers attempted to support the students was the developing a close and supportive relationships. For many of those participants in this review, the maintaining of close relationships with their students was a critical step in supporting them through their mental crisis. Many participants noted that these supportive trusting relationships increased help-seeking behaviour (68). Having established a trusting relationship, many teachers were then able to make the classroom a safe learning environment, one in which was designed to make the student feel more inclusive and less isolated (69, 70). It is evident that this approach has been strongly linked to improved social-emotional health among adolescents (71).

Teachers clearly understood the conflicts imposed by the educational system in support students with a mental health issue. Most of the studies reviewed identified the constraints in the educational system that helped or hindered teachers from providing mental health support to their students. From referring to counselling services, or insisting the student’s parents remove the student from school until their mental health issue had resolved or implement screening programmes to identify those students at risk (72). Yet, it was evident from this review that teachers may be the first individuals to recognise a mental health issue in the student, however, this was often identified in ways other than through mental health screening, such as an abrupt change in behaviour. This created a dilemma for the teachers concerned especially in terms of what their role as a teacher was and the how this might impact on the mental well-being of the student. This impacted significantly in recognising and responding appropriately to the student and more so their ability to support the student. However, there are many factors which undermined the teachers’ confidence and willingness to play an active role in supporting students, such as their own negative emotional experiences to mental health issues and the tension between their personal and professional identity (58). In addition, not having enough resources or time or the inflexibility of the educational system towards mental health issues was also a major concern for teachers as they attempted to balance multiple competing priorities within the school (73). For example, large class sizes also posed a significant barrier to building meaningful relationships with students, identifying at-risk students, and providing timely support for those struggling with mental health issues.

There were three limitations to this review. First, the review was confined to studies reported in English and Chinese and therefore, secondary teachers’ experiences of supporting mental health issues may not be fully representative. However, we synthesised the findings from nine different countries and noted similarities between them. Second, there was a limitation regarding dependability inasmuch that the ConQual score categorised these studies as having a moderate level of dependability only because most of the studies did not include a statement about the authors’ influence over research or vice versa (Q7), which dropped the overall score for dependability. This might be a moot point given the remaining questions in the appraisal process adequately describe the study’s main methodological approach.

## 7 Conclusions

This review revealed the experiences of secondary school teachers in supporting mentally unwell students. As mentioned earlier, teachers felt uncertain feelings and felt helpless to intervene effectively. Teachers’ practices were based on their relationship with the students. However, teachers faced conflicts imposed by the education system when offering support to students, and they may develop compassion fatigue as a result of the above experiences. This could have implications for future educational practice and research in the following ways: The introduction of a psycho-therapeutic support program, such as psychotherapy, by experienced mental health professionals. Teachers could use this approach to develop strategies based on their experiences supporting mentally unwell students individually or collectively. Investigating secondary school teachers’ perceptions, understandings, and experiences in supporting mentally unwell students from more diverse communities. Developing education and/or training programs that raise awareness to recognise and assess culturally related risks.

## Data Availability

The original contributions presented in the study are included in the article/supplementary material. Further inquiries can be directed to the corresponding author.
